# Antiviral Drug–Resistant Influenza B Viruses Carrying H134N Substitution in Neuraminidase, Laos, February 2016

**DOI:** 10.3201/eid2304.161876

**Published:** 2017-04

**Authors:** Tatiana Baranovich, Phengta Vongphrachanh, Pakapak Ketmayoon, Thongchanh Sisouk, Khampheng Chomlasack, Viengphone Khanthamaly, Ha Thuy Nguyen, Vasiliy P. Mishin, Henju Marjuki, John R. Barnes, Rebecca J. Garten, James Stevens, David E. Wentworth, Larisa V. Gubareva

**Affiliations:** Carter Consulting, Inc., Atlanta, Georgia, USA (T. Baranovich);; World Health Organization Collaborating Center for Surveillance, Epidemiology and Control of Influenza, Atlanta (T. Baranovich, V. Khanthamaly, H.T. Nguyen, V.P. Mishin, H. Marjuki, J.R. Barnes, R.J. Garten, J. Stevens, D.E. Wentworth, L.V. Gubareva);; Centers for Disease Control and Prevention, Atlanta (T. Baranovich, V. Khanthamaly, H.T. Nguyen, V.P. Mishin, H. Marjuki, J.R. Barnes, R.J. Garten, J. Stevens, D.E. Wentworth, L.V. Gubareva);; National Center for Laboratory and Epidemiology, Vientiane, Laos (P. Vongphrachanh, P. Ketmayoon, T. Sisouk, K. Chomlasack);; World Health Organization Emerging Disease Surveillance and Response Unit, Vientiane, Laos (P. Ketmayoon);; Battelle Memorial Institute, Atlanta (H.T. Nguyen)

**Keywords:** influenza B viruses, H134N substitution, mutation, neuraminidase, viruses, antiviral drug–resistant, Laos, antimicrobial resistance, influenza

## Abstract

In February 2016, three influenza B/Victoria/2/87 lineage viruses exhibiting 4- to 158-fold reduced inhibition by neuraminidase inhibitors were detected in Laos. These viruses had an H134N substitution in the neuraminidase and replicated efficiently in vitro and in ferrets. Current antiviral drugs may be ineffective in controlling infections caused by viruses harboring this mutation.

Influenza B viruses cause annual epidemics and contribute to ≈30% of influenza-associated deaths among children in the United States (*1*). Two lineages, B/Victoria/2/87 and B/Yamagata/16/88, have been co-circulating globally in recent years (*2*,*3*). Neuraminidase (NA) inhibitors (NAIs) are the only drugs available for treating influenza B virus infections, but NA mutations that emerge during treatment or due to natural variance can diminish the usefulness of NAIs.

## The Study

For this study, the National Center for Laboratory and Epidemiology in Vientiane, Laos, a member of the World Health Organization Global Influenza Surveillance and Response System, provided influenza A and B viruses to the World Health Organization Collaborating Center at the Centers for Disease Control and Prevention (CDC) in Atlanta, Georgia, USA; the viruses had been collected during October 1, 2015–February 29, 2016. We propagated the viruses and then used the CDC standardized NA inhibition assay to assess their susceptibility to NAIs (*4*). Compared with the median 50% inhibitory concentration (IC_50_) values for B-Victoria lineage viruses, IC_50_ values for 2 of the 24 B-Victoria lineage viruses, B/Laos/0406/2016 and B/Laos/0525/2016, were elevated for zanamivir (129- to 158-fold), oseltamivir (4-fold), peramivir (72- to 74-fold), and laninamivir (41- to 42-fold) (Table 1). These results were interpreted as highly reduced inhibition by zanamivir, normal inhibition by oseltamivir, and reduced inhibition by peramivir and laninamivir (Table 1) (*5*). 

**Table 1:** Neuraminidase inhibitor susceptibility of influenza B viruses isolated from human respiratory specimens. Laos, 2016*

Virus isolate	NA amino acid change§	Mean IC_50_ ± SD, nmol/L (fold change)†‡					Date specimen collected	GISAID accession no.
Zanamivir	Oseltamivir¶	Peramivir	Laninamivir
B/Laos/0080/2016	H134	1.09 ± 0.16 (1)	14.48 ± 1.76 (1)	0.36 ± 0.05 (1)	1.15 ± 0.02 (1)		14 Jan	EPIISL 222862
B/Laos/0406/2016	H134N	148.36 ± 14.40 (**129**)	37.87 ± 1.96 (4)	31.09 ± 3.70 (**74**)	62.43 ± 4.66 (**42**)		9 Feb	EPIISL 230596
B/Laos/0525/2016	H134N	176.03 ± 11.14 (**158**)	37.55 ± 5.60 (4)	30.25 ± 2.90 (**72**)	60.12 ± 2.38 (**41**)		15 Feb	EPIISL 230599
B/Laos/0654/2016	H134N	151.95 ± 16.30 (**138**)	35.06 ± 5.08 (4)	31.29 ± 0.24 (**75**)	61.53 ± 1.03 (**42**)		25 Feb	EPIISL 230600

*Viruses were isolated and propagated on MDCK cells. Susceptibility was determined using a fluorescence-based neuraminidase (NA) inhibition assay.  †IC_50_ values (NA inhibitor concentration needed to reduce NA activity by 50%) represent mean ± SD from 3 independent experiments.  ‡Fold change compared with the median IC_50_ value determined for influenza B-Victoria lineage viruses (n = 430) that were circulating worldwide during the 2015‒16 influenza season. Median IC_50_ values are 1.11, 9.67, 0.42, and 1.47 nM for zanamivir, oseltamivir, peramivir, and laninamivir, respectively. Bold indicates fold increases that correspond to reduced inhibition (5- to 50-fold) or to highly reduced (>50-fold) inhibition by a NAI, as outlined by the World Health Organization Expert Working Group of the Global Influenza Surveillance and Response System for Surveillance on Antiviral Susceptibility (*5*). §Amino acid residue 134 in influenza type B NA corresponds to residue Q136 in N1 and N2 NA amino acid numbering (*6*). ¶Oseltamivir carboxylate was used in NA inhibition assay.

**Figure 1:**
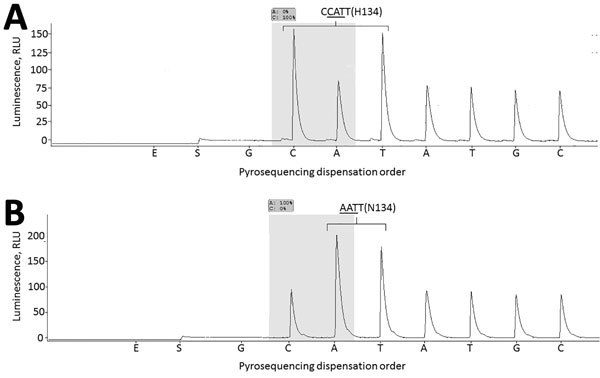
Neuraminidase gene segment (nts 399–497) of influenza B/Laos/0080/2016 virus carrying NA-H134 (A) and B/Laos/0654/2016, NA-N134 (B). RNA extracted from respiratory specimens was used for reverse transcription PCR (RT-PCR) amplification. Two primers, NA-B-242F (5′-CATACCCGCGTTTATCTTGC-3′, forward primer) and NA-B-426Rb (biotin-5′-CTGTCTCCTCTTGTTCCATTGTAG-3′, reverse biotinylated primer) were used in RT-PCR, essentially as described previously (*10*); primer NA-B-378Fs (5′-TGCAAACACTTTGCTTTAAC-3′) was used for pyrosequencing. Underlining indicates nucleotide triplet encoding amino acid residue 134.Shading indicates the nucleotides used to determine the proportion of H134 and N134 neuraminidase variants. Pyrosequencing dispensation order: E-Enzyme mixture; S-substrate mixture; G, C, A and T – nucleotides dGTP, dCTP; dATPαS and dTTP, correspondingly.

This interpretation is useful but obscures the higher median oseltamivir IC_50_ value (9.67 nmol/L vs. 0.42–1.47 nmol/L for other NAIs; Table 1) and the lower potency of oseltamivir in inhibiting NA activity of influenza B viruses (*4*,*7*). Moreover, reports from clinical studies indicate a lesser susceptibility of influenza B viruses to oseltamivir than to zanamivir (*7*–9). Although the laboratory criteria defining clinically relevant NAI resistance are not established, the inhibitory profiles of these 2 viruses suggest resistance to >1 antiviral drugs. NA sequence analysis revealed that both viruses had an amino acid substitution, histidine (H)→asparagine (N), at the highly conserved residue 134 (NA-H134N) (*6*); the presence of H134N in the respiratory specimens was confirmed by pyrosequencing (Figure 1) (*10*). NA-H134Y was previously reported in influenza B virus displaying reduced inhibition by peramivir (*11*). The inhibition profile of influenza B viruses bearing NA-H134N resembles that of influenza A(H1N1) viruses carrying NA-Q136R (residue 134 in influenza B NA corresponds to 136 in N1 numbering) (*12*). Residue 134 (136) has been implicated in the conformational change of the 150-loop, which may adversely affect the interaction between the NA active site and NAIs, especially those containing the guanidyl group (Technical Appendix Figure).

To expand testing, the Laos National Center for Laboratory and Epidemiology provided 40 additional specimens that were positive for B-Victoria lineage virus by real-time reverse transcription PCR (*13*), bringing the total number tested to 64. The specimens were collected during October 2015–April 2016 in Champasack (n = 41), Vientiane (n = 12), Luangprabang (n = 7), and Saravanh (n = 5) Provinces from 28 male and 37 female patients (median age 7 [range 0–67] years). Pyrosequencing revealed NA-H134N in 1 specimen; the respective isolate, B/Laos/0654/2016, displayed the expected NA inhibition profile (Table 1). In total, we found the NA-H134N substitution in 3 (4.6%) of the 65 tested B-Victoria viruses. Analysis of NA sequences deposited to the GISAID database (http://www.gisaid.org) revealed that among 8,601 sequences of influenza B viruses collected worldwide during October 2014–September 2016, only 3 other sequences contained a substitution at H134 (2 harbored H134Y and 1 H134L); the 3 sequences were for B-Victoria lineage viruses.

Epidemiologic data revealed that the NA-H134N viruses were collected from a young woman, a young man, and a 3-year-old girl residing in 2 distant provinces (Table 2). The 3 infections occurred 6–10 days apart in February 2016, and 1 of the patients received medical care for severe acute respiratory illness. No epidemiologic links were identified among the 3 patients infected with the drug-resistant viruses, and patients had no documented exposure to NAIs.

**Table 2:** NA-H134N substitution–containing influenza B viruses that caused confirmed infection among 3 persons, Laos, February 2016*

Virus name, passage history§	Amino acid change in virus genes†										Patient information			
	NA			HA		M1			NS1‡		Age, y/sex	Clinical pres.	Location, province	Date specimen collected
	H134¶	D390		V225		E73	H159		V220					
B/Laos/0406/2016											22/F	SARI	Vientiane	Feb 9
Original	N	D/E		‒		‒	Q		‒					
C2	N	D/E		‒		E/G	Q		‒					
B/Laos/0525/2016											23/M	ILI	Champasack	Feb 15
Original	N	‒		A		‒	Q		‒					
C2	N	‒		A		‒	Q		‒					
B/Laos/0654/2016											3/F	ILI	Champasack	Feb 25
Original	N	‒		‒		‒	Q		I					
C1	N	‒		‒		‒	Q		I					

HA, hemagglutinin; ILI, influenza-like illness; M1, matrix protein 1; NA, neuraminidase; NS1, nonstructural protein 1; SARI, severe acute respiratory illness; –, indicates same amino acid residue as in the consensus sequence of B-Victoria viruses that were circulating in Laos during the 2015‒16 influenza season. †Strain-specific amino acid sequence numbering is used. Sequences for all 8 gene segments were generated using the MiSeq sequencing system (Illumina Inc., San Diego, CA, USA) (*14*) (Technical Appendix Table 1). Data are amino acid differences in protein-coded regions of NA, HA, M1, and NS1 genes compared with the consensus sequence. Not shown: synonymous nucleotide mutations that were common for the NA-H134N viruses: PB1-c93t, PB1-g1930a, and HA-g1520a. In addition, NA-H134N viruses differed from each other by the following synonymous nucleotide mutations: B/Laos/0406/2016 possessed NS1-g345a, B/Laos/0525/2016 possessed NA-t762a, NS1-a561g, and B/Laos/0654/2016 possessed NS2-g186a. ‡Corresponds to position 219 in NS1 protein numbering used by Ma et al. (*15*). §Original indicates specimen collected from patient; C1 and C2 indicates virus propagated once or 2 times on MDCK cells. ¶Presence of the NA-H134N substitution in the original respiratory specimens was confirmed by a pyrosequencing assay (see Figure 1) (Technical Appendix Table 2).

The 3 drug-resistant viruses were genetically similar to other B-Victoria lineage viruses circulating in Laos during 2015‒2016. Besides having the NA-H134N amino acid substitution, these viruses also shared the M1-H159Q amino acid substitution not identified in other virus sequences (Table 2). Also, these viruses have 3 synonymous nucleotide mutations: PB1-c93t, PB1-g1930a, and HA-g1520a. In addition, B/Laos/0406/2016, B/Laos/0525/2016, and B/Laos/0654/2016 harbored substitutions NA-D390D/E, HA-V225A, and NS1-V220I, respectively (Table 2). An analysis of influenza B NS1 sequences available in the GISAID database (as of September 12, 2016) indicated that NS1-V220I is rare, present in only 7 (0.1%) of 10,405 sequences. Taken together, the geographic distance between the sites where the drug-resistant viruses were collected and the differences in their genomes point toward the possibility of influenza NA-H134N viruses circulating in Laos communities.

**Figure 2:**
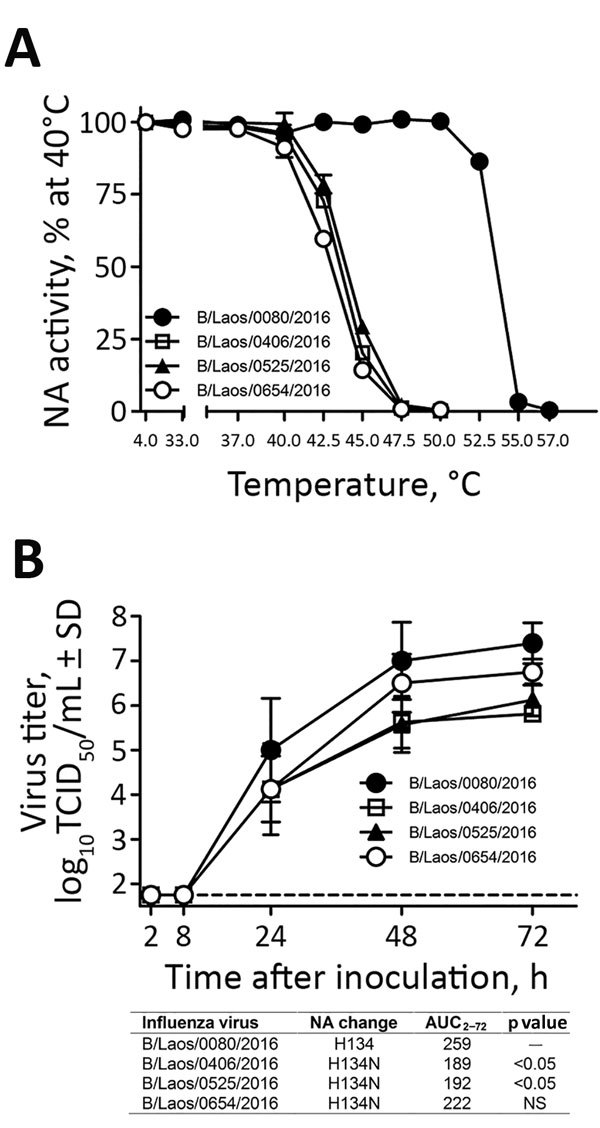
Characterization of influenza B viruses detected in Laos, February 2016. A) Thermostability of neuraminidase (NA) determined after viruses were incubated for 15 min at 4°C or at 30°C–57°C. NA enzyme activity was determined by a fluorescence-based assay (*4*). B) Replication kinetics of influenza B viruses in fully differentiated human primary NHBE cells that were inoculated with the designated viruses (multiplicity of infection 0.001). Apical washes were taken at indicated times after inoculation, and virus titers were determined on MDCK cells. The area under the virus titer curve from 2 to 72 h after inoculation (AUC_2–72_) was determined and compared with that of the control virus by repeated-measures analysis of variance with the Dunnett posttest, using GraphPad Prism 5 software (GraphPad Software, La Jolla, CA, USA). Dashed line represents the limit of detection of the assay (1.75 log_10_ 50% tissue culture infectious dose [TCID_50_/mL]). Values shown are means and SDs from 2 independent experiments performed in duplicates (n = 4). Error bars represent SDs. NS, not significant.

Results of the NA inhibition assay showed that NA-H134N impairs binding of NAIs to the active site of the enzyme. To determine whether this change also affects other properties (e.g., thermostability) of the enzyme, we incubated 3 H134N viruses at elevated temperatures for 15 min and then assessed their NA activity (Figure 2, panel A). The H134N substitution reduced the thermostability of the enzyme. This was evident from the undetectable activity levels starting at 47.5°C, which was 7.5°C lower than that for the control virus, B/Laos/0880/2016, with H134 (p<0.001) (Figure 2, panel A).

To assess the replicative fitness of NA-H134N viruses, we used primary human differentiated normal human bronchial epithelial (NHBE) cells, a cell culture system that morphologically and functionally recapitulates the human airway. The NA-H134N viruses displayed ≈1–2 log_10_ lower titers at 24–72 h after inoculation (Figure 2, panel B). Although, the virus yield reduction (area under the curve) was evident for 2 of the NA-H134N viruses (AUC_2_–_72_ [p<0.05]) (Figure 2, panel B), the difference was not statistically significant for B/Laos/0654/2016 (Figure 2, panel B). The growth kinetics data in differentiated NHBE cells indicate an attenuated phenotype for NA-H134N viruses in vitro. Unlike the other 2 drug-resistant viruses, B/Laos/0654/2016 had substitution NS1-V220I, which resides at the recently discovered second RNA binding site of the NS1 protein of influenza B viruses (*15*). This finding suggests a possible compensatory effect of NS1-V220I on the in vitro replicative capacity of B/Laos/0654/2016.

We assessed the replicative fitness of drug-resistant B/Laos/0654/2016 in three 4- to 6-month-old male ferrets (*Mustela putorius furo*) (Triple F Farms, Sayre, PA, USA) that were serologically negative by HI assay for currently circulating influenza A(H1N1)pdm09, A(H3N2), and B viruses. At 48 h after inoculation with virus (10^4^ 50% tissue culture infectious dose/mL), ferrets displayed fever (>1.5°C above baseline) that lasted 21.8 ± 5.1 h on average. Virus shedding lasted 6 days; nasal wash virus titers, which were determined daily, were 4.2 ± 0.4; 6.0 ± 0.2; 4.8 ± 0.4, 4.7 ± 0.4, 4.7 ± 0.6, and 2.8 ± 0.4 log_10_ 50% tissue culture infectious doses/mL, respectively. These data suggest that the drug-resistant virus can replicate to high titers in the upper respiratory tract of ferrets and induce persistent fever.

## Conclusions

In February 2016, we detected 3 influenza B viruses in Laos bearing a rare NA-H134N substitution. Current antiviral medications may not effectively control infections caused by such viruses. Virus harboring NA-H134N and NS1-V220I replicated efficiently in NHBE cells and in the ferret upper respiratory tract. Studies to ascertain the effect of NA-H134N and NS1-V220I on influenza B virus virulence and transmissibility in a mammalian host are needed.

Technical Appendix Quantification of a proportion of H134 and N134 neuraminidase variants in respiritory specimens harboing influenza B viruses collected in Laos, February 2016.
