# Supplementary material for: Antiviral Drug–Resistant Influenza B Viruses Carrying H134N Substitution in Neuraminidase, Laos, February 2016
Source: Emerg Infect Dis. 2017 Apr;23(4):686–90. doi: 10.3201/eid2304.161876 (PMC5367415; doi:10.3201/eid2304.161876)
Supplement: Technical Appendix — Quantification of a proportion of H134 and N134 neuraminidase variants in respiritory specimens harboing influenza B viruses collected in Laos, February 2016. [file 16-1876-Techapp-s1.pdf]

Article DOI: http://dx.doi.org/10.3201/eid2304.161876

Antiviral Drug–Resistant Influenza B Viruses Carrying H134N Substitution in Neuraminidase, Laos, February 2016

Technical Appendix

Technical Appendix Table 1. Accession numbers for the genome sequences deposited into the GISAID database

Virus name	Collection Date	NA amino acid change	Genome Segment							
			PB2	PB1	PA	HA	NP	NA	MP	NS
B/Laos/0080/2016	2016-01-14	None	EPI765461	EPI765462	EPI765460	EPI765464	EPI765457	EPI765463	EPI765459	EPI765458
B/Laos/0406/2016	2016-02-09	H134N	EPI775445	EPI775446	EPI775444	EPI775448	EPI775441	EPI775447	EPI775443	EPI775442
B/Laos/0525/2016	2016-02-15	H134N	EPI765556	EPI765557	EPI765555	EPI765559	EPI765552	EPI765558	EPI765554	EPI765553
B/Laos/0654/2016	2016-02-25	H134N	EPI775437	EPI775438	EPI775436	EPI775440	EPI775433	EPI775439	EPI775435	EPI775434

Technical Appendix Table 2. Quantification of a proportion of H134 and N134 neuraminidase variants in respiritory specimens harboring influenza B viruses collected in Laos, February 2016*

Virus	NA amino acid	Mean (%) ± SD	
		H134	H134N
B/Laos/0080/2016	H134	99.6 ± 0.5	0.4 ± 0.5
B/Laos/0406/2016	H134N	0.7 ± 0.6	99.3 ± 0.6
B/Laos/0525/2016	H134N	1.6 ± 0.7	98.4 ± 0.7
B/Laos/0654/2016	H134N	0	100.0

*Pyrosequencing analysis in the allele quantification mode was conducted on the respective respiratory specimens in triplicate.

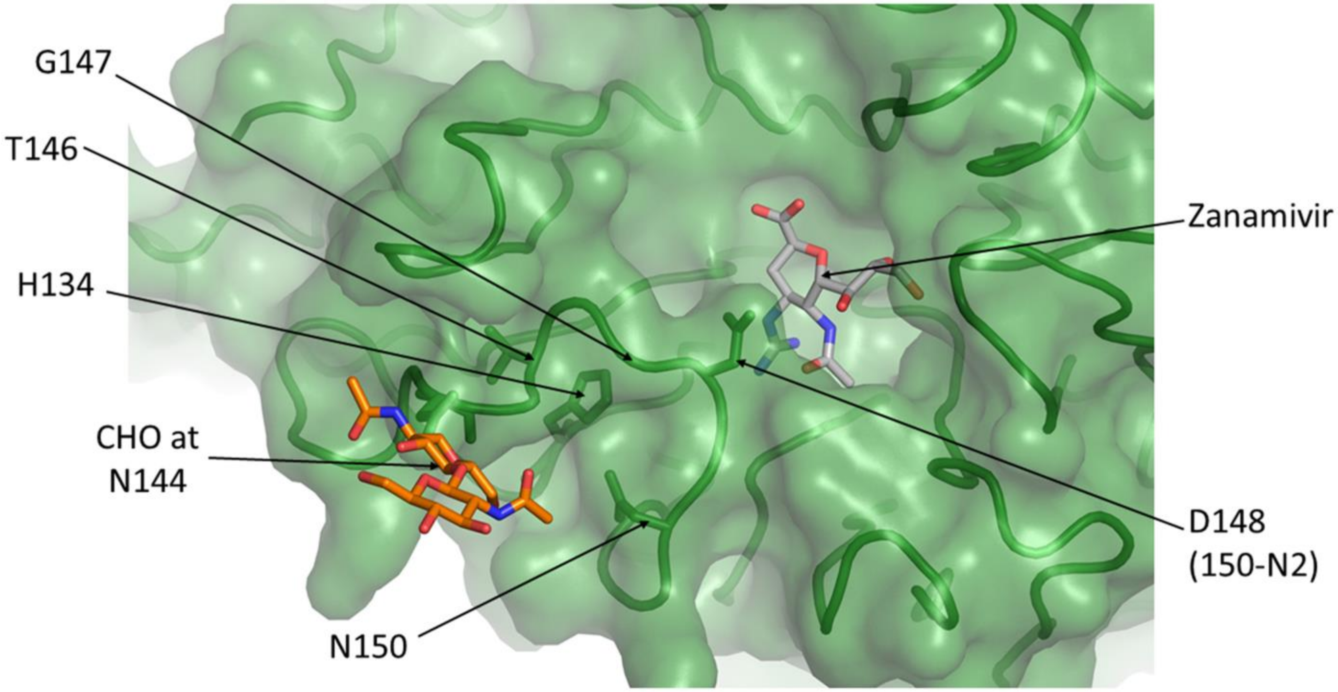

Technical Appendix Figure. Structure of the active site of B/Brisbane/60/2008 (Victoria lineage) neuraminidase with the bound neuraminidase inhibitor zanamivir; Protein Data Bank code 4cpn.
